# Safety Planning With Patients Admitted to the Acute Hospital – Current Practice and Future Directions

**DOI:** 10.1192/bjo.2025.10662

**Published:** 2025-06-20

**Authors:** Alice Reid, Sarah Piper, Merryn Anderson, Jessica Lynch

**Affiliations:** Devon Partnership Trust, Exeter, United Kingdom

## Abstract

**Aims:** Safety planning has been identified as best practice for suicide prevention and is used to support patient who are at a high risk of suicide. A key aspect of safety planning is collaborative involvement with the patient and their family/carers.

Aims were to audit current compliance with safety planning standards for patients admitted to an acute hospital under the Exeter Psychiatry Liaison Team.

**Methods:** A snapshot audit was carried out for patients that had been admitted to Exeter Liaison Psychiatry caseload as inpatients over a two-month period. 25% of patients were reviewed, the patients being selected through a random number generator to ensure minimal bias. Initial assessment and discharge summary documents were reviewed, and data collected onto an Excel spreadsheet to record compliance with three standards.

Standard 1: Safety plan recorded – target compliance 95%.

Standard 2: Documentation that safety plan was collaboratively generated – target compliance 95%.

Standard 3: Documentation that patients were provided with a written copy of the safety plan.

**Results:** Data was collected from 25% of inpatients (n=29). Following initial assessment, safety plans were created with 69% of patients, 15% of these were documented to be co-created, and 0% were evidence to be provided in writing. At point of discharge, safety plans were created for 52% of patients, with 40% evidence to be co-created, and 33% were evidenced to be provided in writing. Duration of time under Liaison Psychiatry varied from 0–54 days, 35% of patients were discharged to a psychiatric admission and 38% discharged to their usual place of residence.

Discussion: There are clear gaps to collaborating with patients to create safety plans, with minimal evidence they are being provided in writing. This could be impacted by the nature of the workload of the Liaison Psychiatry department, and the unpredictability of awaiting availability of mental health beds. Patients awaiting psychiatric beds may have been too unwell to engage with safety planning. There should be consideration for how to keep this document live and accessible by patients, their regular clinicians and those who may encounter the patient at a time of crisis (GP, mental health teams, liaison teams, emergency department staff and emergency workers).

**Conclusion:** Prolonged inpatient admission due to either mental health or physical health reasons provides a good opportunity to engage patients with safety planning, an opportunity which is not being utilised within this Liaison Psychiatry department. Within this department there needs to be further uptake of engagement in safety planning.

